# Acquisition matters - how do scan parameters affect apparent diffusion coefficient estimates in pediatric rhabdomyosarcoma

**DOI:** 10.1007/s00247-025-06263-w

**Published:** 2025-06-10

**Authors:** Cyrano Chatziantoniou, Roelof van Ewijk, Madeleine Adams, Patrizia Bertolini, Gianni Bisogno, Amine Bouhamama, Pablo Caro-Dominguez, Valérie Charon, Ana Coma, Rana Dandis, Christine Devalck, Giulia De Donno, Andrea Ferrari, Marta Fiocco, Soledad Gallego, Chiara Giraudo, Heidi Glosli, Simone A. J. ter Horst, Meriel Jenney, Willemijn M. Klein, Alexander Leemans, Julie Leseur, Henry C. Mandeville, Kieran McHugh, Johannes H. M. Merks, Veronique Minard-Colin, Salma Moalla, Carlo Morosi, Daniel Orbach, Lil-Sofie Ording Müller, Erika Pace, Pier Luigi Di Paolo, Katia Perruccio, Lucia Quaglietta, Marleen Renard, Rick R. van Rijn, Antonio Ruggiero, Sara I. Sirvent, Reineke A. Schoot, Alberto De Luca

**Affiliations:** 1 University Medical Center Utrecht, Utrecht, Heidelberglaan 100, 3584 CX Netherlands; 2 Princess Máxima Center, Utrecht, Netherlands; 3 Princess Máxima Center, Utrecht, Netherlands; 4 Noah’s Ark Children’s Hospital for Wales, Cardiff, United Kingdom; 5 University Hospital of Parma, Parma, Italy; 6 University of Padua, Padova, Italy; 7 Centre Léon Bérard, Lyon, France; 8 Hospital Universitario Virgen del Rocío, Seville, Spain; 9 Centre Hospitalier Universitaire de Rennes, Rennes, France; 10 Vall d’Hebron Hospital Universitari, Barcelona, Spain; 11 Queen Fabiola Children’s University Hospital, Brussels, Belgium; 12 Fondazione IRCCS Istituto Nazionale dei Tumori, Milan, Italy; 13 Leiden University Medical Center, Leiden, Netherlands; 14 Oslo University Hospital, Oslo, Norway; 15 Wilhelmina Children’s Hospital, Utrecht, Netherlands; 16 Radboud University Nijmegen Medical Centre, Nijmegen, Netherlands; 17 Centre Eugène Marquis, Rennes, France; 18 Royal Marsden Hospital, London, United Kingdom; 19 Great Ormond Street Hospital for Children NHS Foundation Trust, London, United Kingdom; 20 University Medical Center Utrecht, Utrecht, Netherlands; 21 Institut Gustave Roussy, Villejuif, France; 22 Institute Curie, Paris, France; 23 Royal Marsden NHS Foundation Trust, London, United Kingdom; 24 Institute of Cancer Research, London, United Kingdom; 25 Bambino Gesù Children’s Hospital, Rome, Italy; 26 Azienda Ospedaliera di Perugia, Perugia, Italy; 27 Department of Hemato-Oncology and Cell Therapy, Santobono-Pausilipon Children’s Hospital, Naples, Italy; 28 Universitair Ziekenhuis Leuven, Leuven, Belgium; 29 Department of Radiology and Nuclear Medicine, Amsterdam UMC, University of Amsterdam, Amsterdam, Netherlands; 30 Catholic University of the Sacred Heart, Milan, Italy; 31 Hospital Infantil Universitario Niño Jesús, Madrid, Spain

**Keywords:** Adolescent, Child, Diffusion magnetic resonance imaging, Rhabdomyosarcoma

## Abstract

**Background:**

The apparent diffusion coefficient (ADC) derived from diffusion-weighted imaging (DWI) is a potential biomarker for treatment response in pediatric rhabdomyosarcoma. Due to its rarity, investigations into this marker require multicenter approaches, which can result in variability in acquisition parameters.

**Objective:**

To evaluate the impact of different acquisition parameters on ADC estimates in a multicenter dataset of rhabdomyosarcoma patients.

**Materials and methods:**

We included 114 pediatric and adolescent rhabdomyosarcoma patients from 22 treatment centers (195 scans). Median age: 6.0 years (0.3–21.8). We evaluated the impact of voxel size, (number of) *b*-values, and echo time on tumor ADC values. The effect of the highest *b*-value was separately investigated on a subset of scans with five or more *b*-values.

**Results:**

We observed a large variability in key acquisition parameters in the overall cohort, and for individual imaging centers. No parameter showed a significant effect on ADC estimates of the whole cohort when corrected for multiple-comparisons. Decreasing the highest *b*-value within the same acquisition caused ADC to decrease on average by 2.8% per 100 s mm^-2^. Differing *b*-values between scans at diagnosis and treatment response yielded significant changes in the longitudinal ADC for each patient (*P*<0.05).

**Conclusion:**

While we observed wide variation of acquisition parameters within a multicenter cohort, this did not lead to significant cross-sectional differences of tumor ADC. However, we found that modifying the highest *b*-value between baseline and follow-up can impact longitudinal ADC estimates. As such, we recommend the highest *b*-value to remain constant.

This retrospective study was reviewed and approved by the Internal Review Board (UMC Utrecht, reference ID: 18-412).

**Graphical Abstract:**

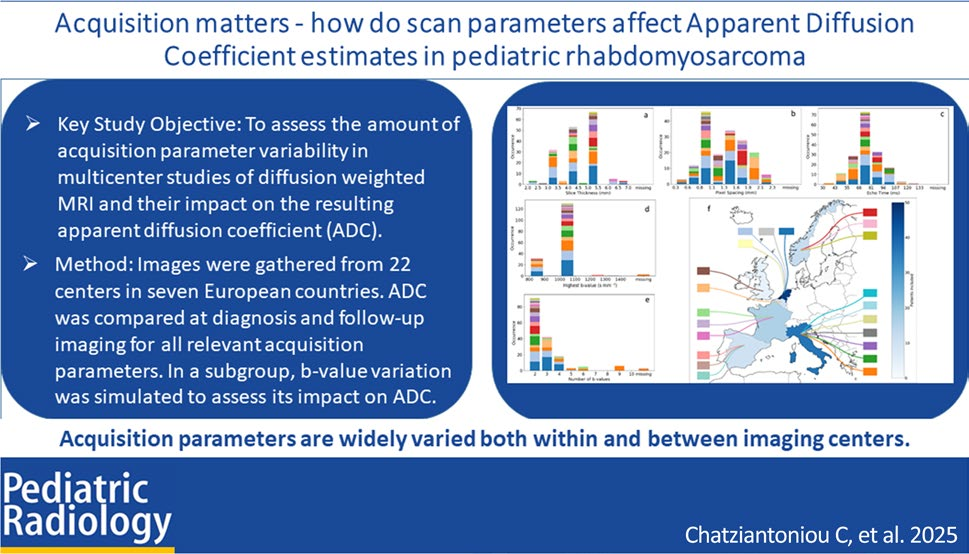

**Supplementary Information:**

The online version contains supplementary material available at 10.1007/s00247-025-06263-w.

## Introduction

Rhabdomyosarcoma is an aggressive soft-tissue tumor that to date lacks a reliable early biomarker for treatment response [1]. Currently, event-free survival and overall survival are used as outcomes in clinical trials; this however takes 7–10 years to achieve. For the introduction of new therapies, this is too long and hampers improvement of treatment and outcome in this rare disease. One promising early biomarker for treatment response is the apparent diffusion coefficient (ADC), which has been shown to be related to cellularity in multiple types of tumors [2–6] and has been linked to treatment response in other soft-tissue tumors [7]. For this reason, diffusion-weighted imaging (DWI), from which the ADC is derived, has been suggested as a modality of interest for determining treatment response [8–12].

A major challenge in implementing a DWI-based marker is its sensitivity to variation in acquisition parameters [13, 14]. This sensitivity may hinder the comparability of ADC values among scans obtained at different treatment centers [15]. Because rhabdomyosarcoma has an incidence of about five cases per million children, most studies rely on a multicenter approach to achieve an adequate sample size [16, 17]. Due to the variability in hardware and software across sites, multicenter studies of rhabdomyosarcoma can potentially vary widely in how data is acquired. Furthermore, imaging in rhabdomyosarcoma often consists of data from several body parts, which intrinsically requires different acquisition parameters optimized for each region.

In this study, we evaluate the effect of acquisition parameters on ADC values in a large retrospective cohort of pediatric and young adolescent rhabdomyosarcoma patients [18]. Particularly, we look at the cross-sectional effect of parameters and the effect of parameter mismatch among longitudinal acquisitions on the value of ADC change as a biomarker. We furthermore compare ADC variability between tumor and healthy tissue.

## Methods

### Patients

For this retrospective analysis, we included pediatric and adolescent patients from 22 centers in seven countries with histologically confirmed rhabdomyosarcoma. The patients all had macroscopic residual disease after initial surgery (Intergroup Rhabdomyosarcoma Study Group III/IV) and were treated according to the European Paediatric Soft Tissue Sarcoma Study Group (EpSSG) rhabdomyosarcoma 2005 [19, 20] or the MTS 2008 protocols [21]. Written consent was obtained from all patients and/or their parents/caretakers. The scans were available from the aforementioned institutional review board–approved international studies. This retrospective study was reviewed and approved by the Internal Review Board (UMC Utrecht, reference ID: 18–412).

### Imaging

Institutional protocols were used for image acquisition since no established acquisition guidelines for DWI were available at the time of scanning. Imaging took place at diagnosis and after 2–4 cycles of chemotherapy (each cycle lasted 3 weeks).

Pseudonymized imaging data was collected on a central platform developed as part of the Quality and Excellence in Radiotherapy and Imaging for Children and Adolescents with Cancer across Europe in Clinical Trials (QUARTET) initiative of the International Society of Pediatric Oncology (SIOP) Europe [22].

### Preprocessing

Pre-computed ADC maps were derived from the scanner vendor software wherever possible. When these maps were not available, but DWI data was present, we computed ADC maps from the DWI data using in-house developed software written in MATLAB (MathWorks, Natick, MA, version 2022a). ADC was calculated by using a mono-exponential fit which was solved via Gaussian elimination.

### Acquisition parameter extraction

We selected the following acquisition parameters of interest: echo time, number of *b*-values used for the DWI acquisition, highest *b*-value, slice thickness, and voxel size. These parameters were selected based on a phantom study that previously evaluated their impact on ADC [14]. We then extracted these data from the Digital Imaging and Communications in Medicine (DICOM) headers of DWI and ADC images. When these parameters were not available, an attempt was made to derive them from other sources, such as the acquisition series description. If this was not possible either, the value for that parameter was left blank.

### Quality assessment of scans

Two experienced pediatric radiologists (S.H. and R.R.), with respectively 11 years and 20 years of experience in pediatric radiology, assessed the scans of eligible patients. They evaluated the ADC quality and the possibility of delineating the tumor on available ADC maps. A subjective scale ranging from 1 to 3 was used: 1 for poor or not evaluable examinations, 2 for moderate and evaluable scans, and 3 for good quality imaging. Scans were excluded from further analysis if they had poor quality, or if they were deemed to not have a measurable tumor on the ADC map by the pediatric radiologists.

### Segmentation

All segmentations were performed using in-house developed delineation software, which allowed for the drawing of polygonal regions of interest (ROI). Tumors were segmented on ADC maps by a team consisting of two pediatric radiologists (S.H. and R.R.) and two trained researchers (R.E. and C.C. both with 3 years of experience in working with pediatric images). Initially, one radiologist performed a single-slice delineation, choosing the axial DW slice that provided the best representation of the tumor. This selection was subsequently verified by the other radiologist. To aid in locating the tumor, reference was made to axial T1- and T2-weighted images. Following the single-slice delineation, a multislice segmentation was conducted by the two trained researchers (R.E. and C.C.) on up to two adjacent upper and lower slices; these were then checked by one pediatric radiologist and updated based on their feedback. The resulting multislice segmentations were utilized for subsequent analysis.

The tumors were delineated at the inner edge to minimize partial volume effects. Any necrotic, cystic, or hemorrhagic areas were separately annotated and excluded from the overall ROI both at the initial and multislice segmentations. These segmentation details were based on a prior investigation to minimize variability [23].

**Fig. 1:**
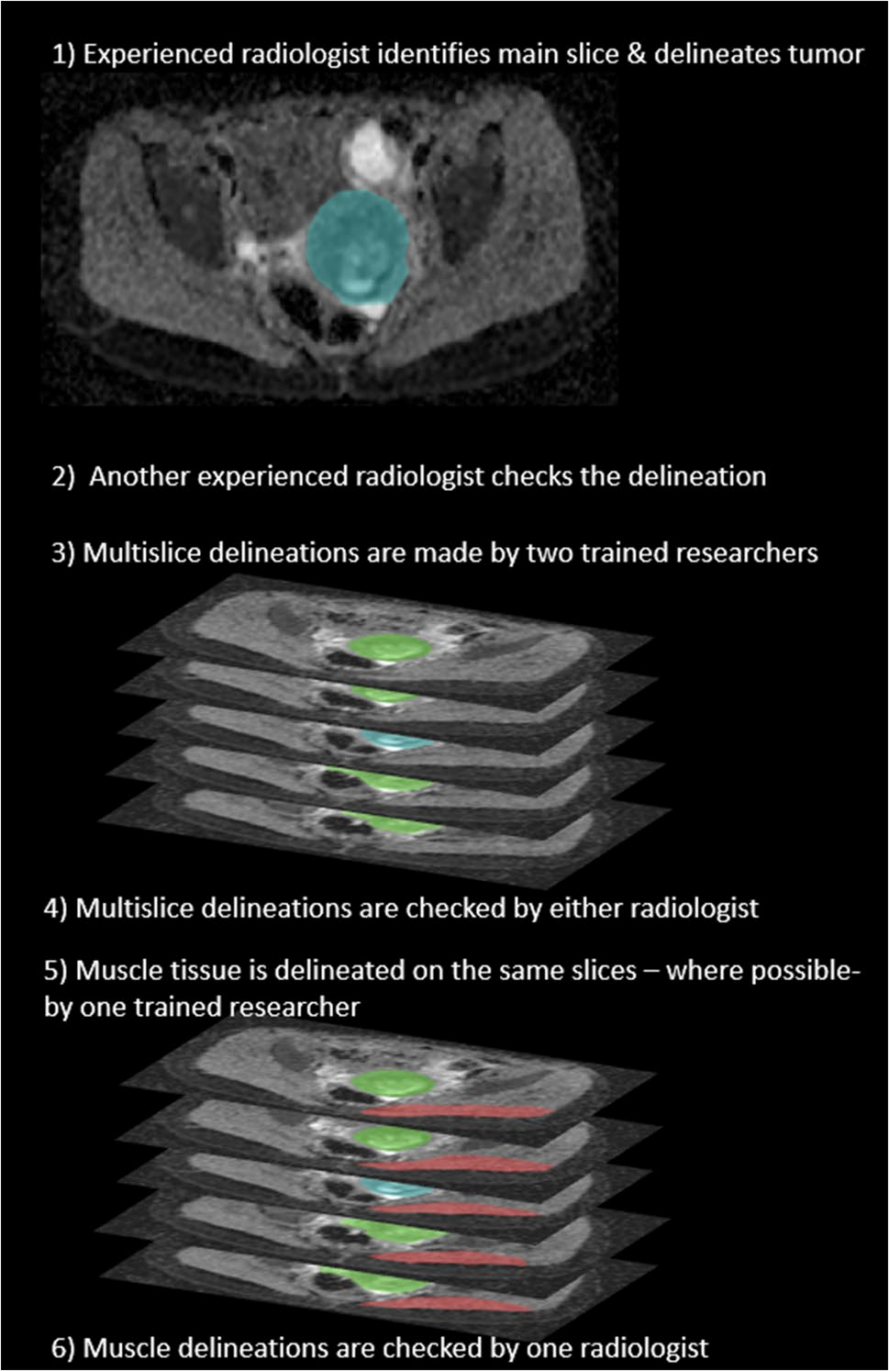
Example of the outline segmentation process for a pediatric rhabdomyosarcoma tumor (*blue*/*green*) and a section of healthy muscle (*red*)

To evaluate whether the impact of the acquisition parameters on ADC estimates are specific to rhabdomyosarcoma or that these can be generalized to other tissues, we also analyzed healthy muscle tissue in a subgroup of patients. A trained researcher (C.C.) evaluated all scans for the presence and image quality of musculature on the slices where the tumor was delineated. Regions of healthy tissue were then segmented on all scans where the image quality was deemed adequate. These segmentations were subsequently reviewed by a pediatric radiologist (S.H.). An overview of the entire segmentation process is shown in Fig. 1.

### Analyses

The median ADC was calculated for all tumor and muscle regions of interest. To assess the ADC variability due to each acquisition parameter of interest, we evaluated the coefficient of variation (CV). This was calculated for the whole cohort and separately for each treatment center.

To examine the impact of this variability on the ADC for each acquisition parameter of interest, the dataset was split into two groups based on the median value of that acquisition parameter. The tumor ADC values between these two groups were then compared by a Mann-Whitney *U* test. If the parameter in question, e.g., the echo time, would impact tumor ADC, then we would expect to observe a significant difference between the two groups. A Bonferroni correction was applied to all tests.

To check for any accidental effects from the previous analysis stemming from dichotomizing continuous variables, a linear regression analysis was performed among the parameters and the ADC for each imaging parameter separately, using them as covariates. These analyses were carried out separately for scans conducted at the time of diagnosis and during the response to chemotherapy.

To investigate the impact of *b*-value choice on ADC, scans with DWI data containing more than five *b*-values were selected. For each of these examinations, ADC was estimated using all unique combinations of *b*-values that included the non-weighted image. The resulting ADC values were grouped and averaged based on the highest *b*-value used in the estimation.

The impact of the highest *b*-value on the longitudinal change in ADC (between diagnosis and treatment response) was subsequently measured. Patients were selected if they had scans with more than five *b*-values at both diagnosis and at response imaging. For these patients, the ADC was estimated with different combinations of *b*-values at both time points, which were then grouped and averaged based on the highest *b*-value. By using either the highest *b*-value or the second highest at both diagnosis and response, the longitudinal change in ADC was then calculated in four different combinations. This scenario was designed to represent situations where a patient undergoes scanning with different acquisition parameters at baseline and after chemotherapy.

For the longitudinal analysis, statistical significance was determined as follows: first the longitudinal change in ADC was obtained for each combination, alongside the propagated uncertainty and the average number of samples. We could then perform a *t*-test for each pair of combinations.

All statistical calculations were performed with scripts written in Python (Wilmington, DE, USA).

## Results

### Patient and scan inclusion

**Fig. 2:**
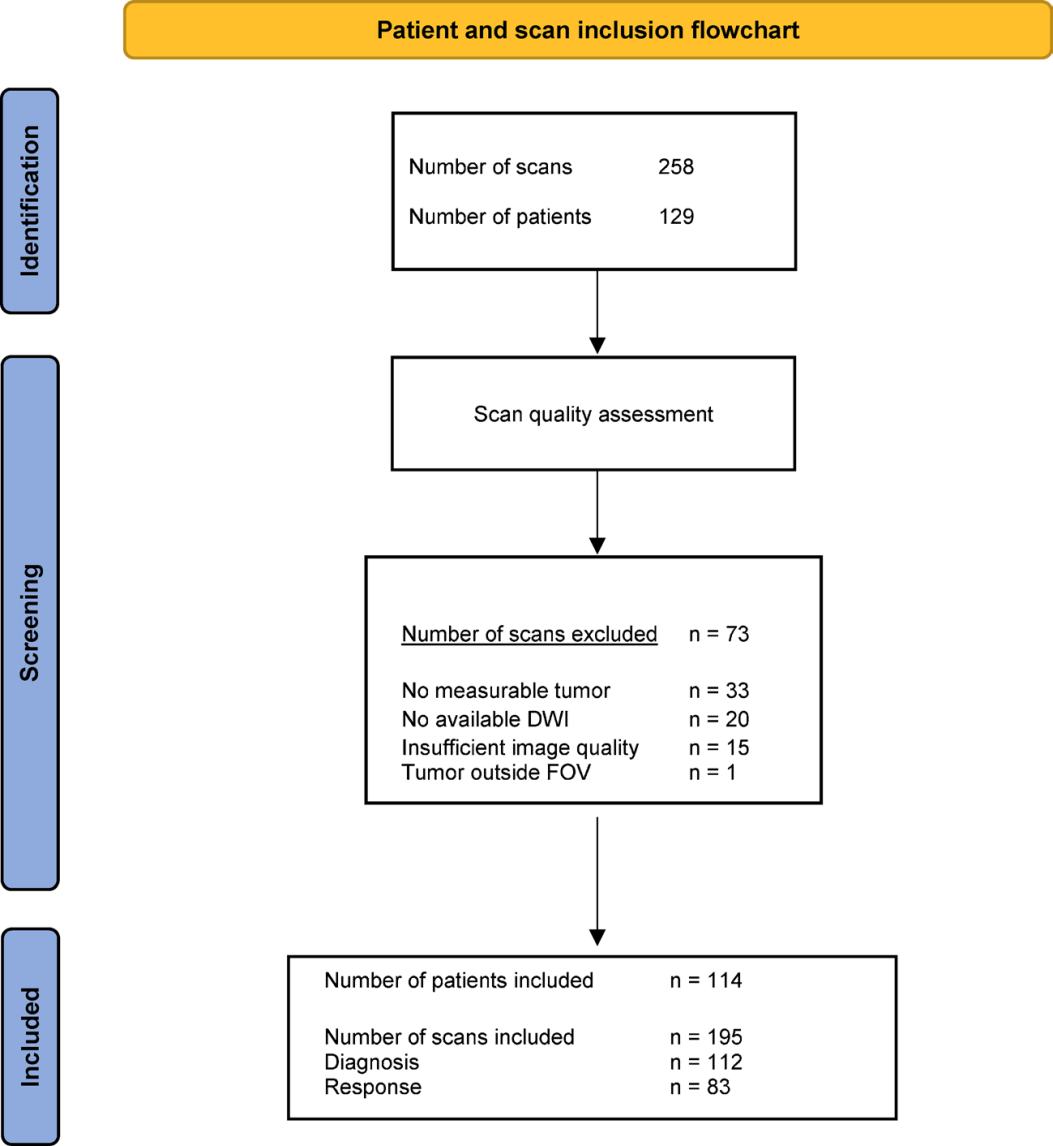
Patient and diffusion-weighted imaging (DWI) selection. *FOV*, field of view

The study included MR examinations from 22 hospitals in seven European countries, with a total of 129 eligible patients and 258 scans. After quality assessment, 195 scans from 114 patients were included in this study. Imaging was excluded when the scan did not contain measurable tumor (33 scans), was of poor quality (15), if there were no DWI data (20), or because the tumor was outside of the field of view (1). Among the 195 included scans, 112 were conducted at the time of diagnosis, while 83 were obtained to evaluate treatment response. Scans were available at both time points for 81 patients. Figure 2 illustrates an overview of the inclusion process. The patient and tumor characteristics of the resulting cohort are shown in Table 1.

**Table 1:** Characteristics of 114 patients affected by rhabdomyosarcoma with at least one diffusion-weighted image of the measurable tumor. Primary tumor sites based on Bisogno 2023 [20]. *GUBP* genitourinary bladder prostate, *GUnoBP* genitourinary non-bladder prostate, *HNnoPM* head and neck non-parameningeal, *HNPM* head and neck parameningeal

	All patients (*N*=134)
Country	
France	16 (11.9%)
Italy	36 (26.9%)
Norway	12 (9.0%)
Spain	10 (7.5%)
The Netherlands	46 (34.3%)
UK	4 (3.0%)
Belgium	10 (7.5%)
Gender	
Female	48 (35.8%)
Male	86 (64.2%)
Age (years)	
Mean (SD)	7.67 (5.67)
Median [min, max]	6.00 [0.300, 21.8]
Site of primary tumor	
Extremities	12 (9.0%)
GUBP	24 (17.9%)
GUnoBP	3 (2.2%)
HNnoPM	17 (12.7%)
HNPM	47 (35.1%)
Orbit	17 (12.7%)
Other site	14 (10.4%)
Histology	
Alveolar	29 (21.6%)
Embryonal	96 (71.6%)
Other	9 (6.7%)
Tumor size	
≤ 5 cm	70 (52.2%)
> 5 cm	64 (47.8%)
Risk group	
Standard	47 (35.1%)
High	51 (38.1%)
Very high - localized	9 (6.7%)
Very high - metastatic	27 (20.1%)

Five patients that were initially included were later removed based on their age. The results of the cohort including these patients are provided as Supplementary Materials 1–6.

### Acquisition parameter variability

Figure 4 illustrates the large variability in the echo time, pixel spacing, highest *b*-value, slice thickness, and number of *b*-values across datasets. This variability is not only present across hospitals, but also within centers and is quantified in Table 2 by means of the coefficient of variation (CV) between centers and within centers. The highest CV is observed for the number of *b*-values. Supplementary Material 7 lists all manufacturer and imaging parameters per center.

**Table 2:** The coefficient of variation (CV) among and within hospitals for various acquisition parameters. The highest CV is seen for the number of *b*-values and the pixel spacing. The CV is calculated by dividing the standard deviation by the mean. It can be seen as the range in which 68.2% (2 sigma) of samples fall, expressed as a fraction of the mean

Parameter	Coefficient of variation (all centers)	Coefficient of variation within centers (median, range)
Echo time	0.18	0.13, 0.007–0.28
Pixel spacing	0.31	0.22, 0.005 - 0.55
Slice thickness	0.22	0.14, 0.08 - 0.32
Number of *b*-values	0.55	0.31, 0–0.58
Highest *b*-value	0.09	0.07, 0–0.18

### Diffusion and tissue type

**Fig. 3:**
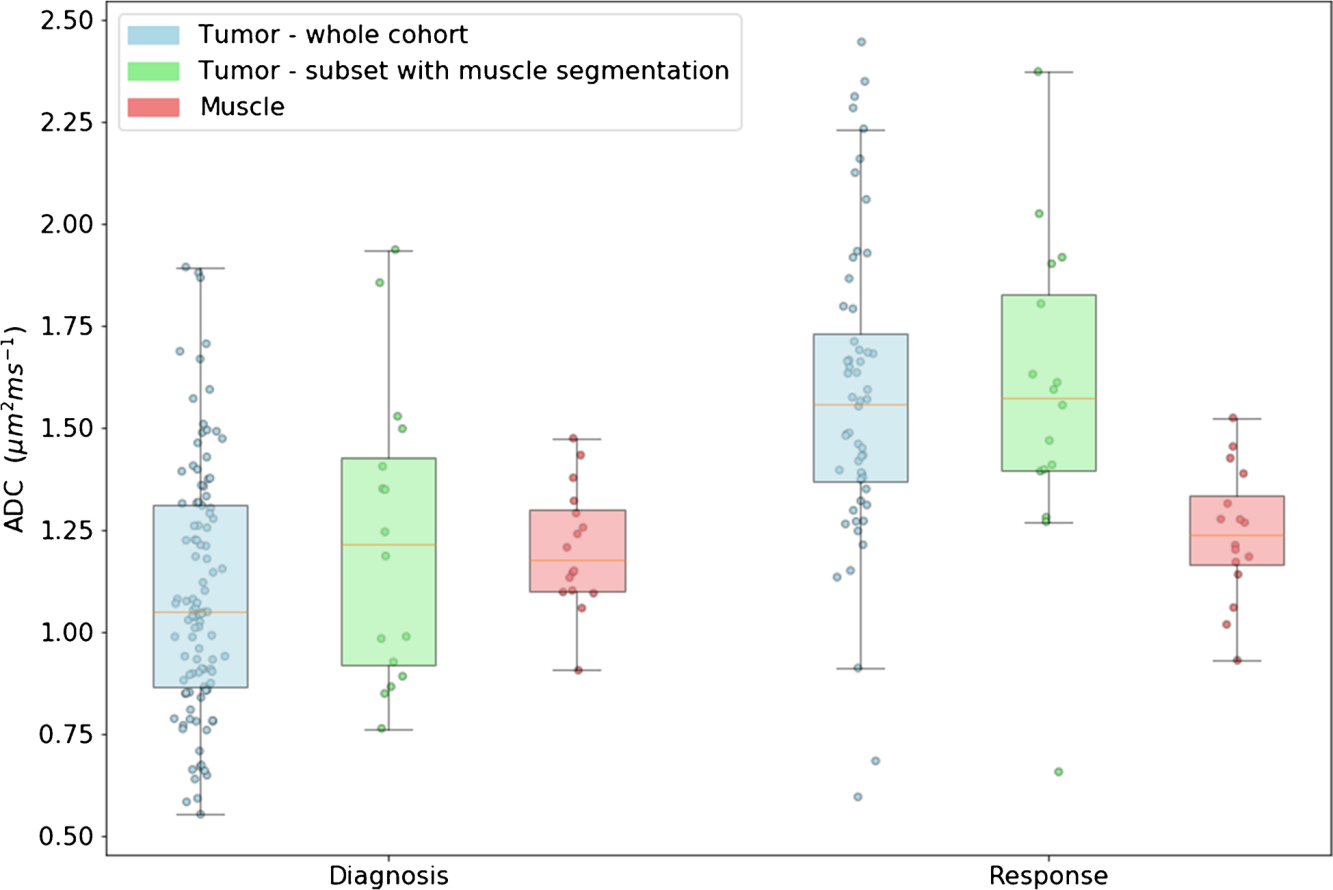
Apparent diffusion coefficient (ADC) estimates of pediatric rhabdomyosarcoma patients. Tumor ADC is shown for a cohort of 114 patients imaged at diagnosis and treatment response (*blue*). For a subset of 16 patients, ADC was also calculated within the musculature (*red*) adjacent to the tumor mass (*green*). The ADC variation is much larger in the tumor tissue than in the unaffected muscle. In fact, the ADC values within the muscle are unaltered between diagnosis and response to chemotherapy, whereas those in the tumors increase over time

We were able to reliably segment healthy muscle tissue both at diagnosis and at response assessment for only 16 patients out of the initial 114 because the image quality of the other subjects was insufficient in the regions with healthy muscle. Figure 3 displays the estimated ADC values for tumor and muscle tissue both at the time of diagnosis and at response assessment.

We find a large range in the ADC values at diagnosis of the entire cohort: 0.55–1.89 µm^2^ ms^−1^, mean 1.09 µm^2^ ms^−1^. We observe a similar range of tumor ADC in the subset for patients with healthy muscle present near the tumor: 0.76–1.93 µm^2^ ms^−1^, mean 1.22 µm^2^ ms^−1^ and a much smaller range for the healthy muscle: 0.91–1.47 µm^2^ ms^−1^, mean 1.20 µm^2^ ms^−1^.

Tumor ADC tends to increase at early response for nearly the whole cohort, with the exception of two patients where ADC decreases. This results in an increased range for tumor ADC. For the whole cohort: 0.60–2.45 µm^2^ ms^−1^, mean 1.58 µm^2^ ms^−1^; for the subset with healthy muscle near the tumor: 0.66–2.37 µm^2^ ms^−1^, mean 1.47 µm^2^ ms^−1^. Healthy muscle ADC is largely similar at early response in terms of range and mean: 0.93–1.53 µm^2^ ms^−1^, mean 1.52 µm^2^ ms^−1^.

The ADC variation is higher in the tumor tissue than in unaffected muscle. Furthermore, the ADC within the muscle has no significant differences between diagnosis and after chemotherapy (*P*=0.53), whereas the tumor ADC significantly increases (*P*<0.01) (Table 3).

**Table 3:** Apparent diffusion coefficient (ADC) estimates of patients with rhabdomyosarcoma. Mean and standard deviation of ADC are shown for tumor tissue and healthy tissue in a cohort of 114 patients and a subset of 16 subjects where healthy muscle tissue was present in-plane with the tumor

	Whole cohort (*N*=117)	Subset (*N*=17)
ADC (μm^2^ ms^−1^) (mean, SD)		
Tumor, diagnosis	1.09 (0.30)	1.23 (0.35)
Healthy muscle, diagnosis	-	1.20 (0.14)
Tumor, response	1.58 (0.38)	1.58 (0.37)
Healthy muscle, response	-	1.24 (0.16)

### Acquisition parameter effect on diffusion

**Fig. 4:**
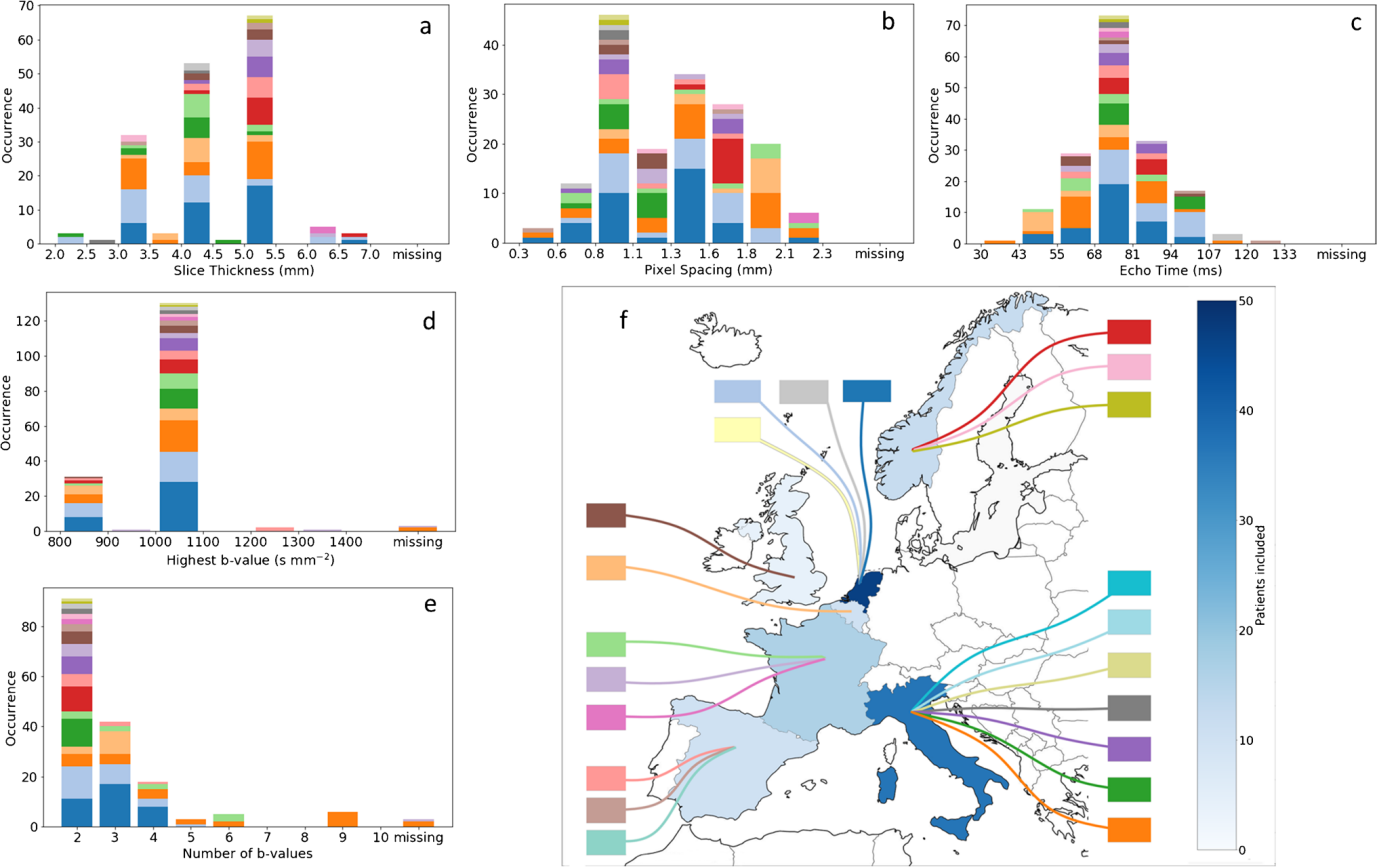
The distribution of the slice thickness (**a**), pixel spacing (**b**), echo time (**c**), highest *b*-value (**d**), and number of *b*-values (**e**) in 268 diffusion-weighted image acquisitions from a retrospective cohort of 134 pediatric rhabdomyosarcoma patients. The acquisitions were made in 22 hospitals across seven European countries (each center shown with a different *color*, **f**). Variability is present for all parameters, both in the entire cohort and within single centers. Additionally, for each parameter, values were missing in 5–10% of scans

**Fig. 5:**
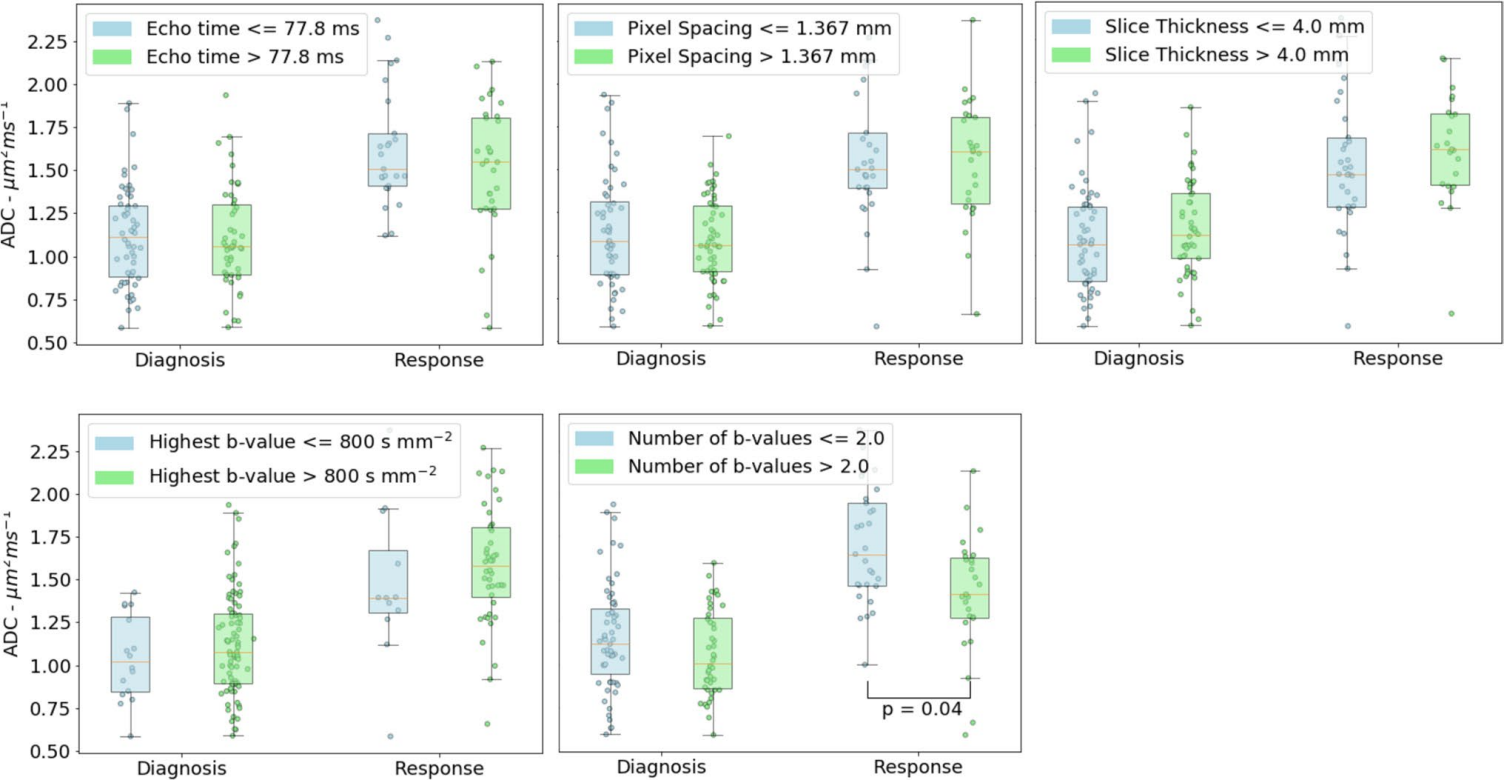
Apparent diffusion coefficient (ADC) estimates of pediatric rhabdomyosarcoma tumors in a cohort of 114 patients imaged at diagnosis and after chemotherapy. The ADC values are shown in groups split across the median for the acquisition parameters: echo time (**a**), pixel spacing (**b**), slice thickness (**c**), highest *b*-value (**d**), and number of *b*-values. The ADC only significantly differs, based on a Mann-Whitney *U* test among the groups at response assessment measurements with respect to the number of *b*-values. When a Bonferroni correction is applied to account for the multiple parameters that were tested, this significance disappears

The effect of various parameters on ADC was investigated for all scans in the cohort (Fig. 4). When dividing the ADC estimates based on the median values of the echo time, pixel spacing, highest *b*-value, and slice thickness, no statistically significant differences were found in the estimated ADC either at diagnosis or after chemotherapy (Fig. 5).

### *B*-value effect on diffusion

**Fig. 6:**
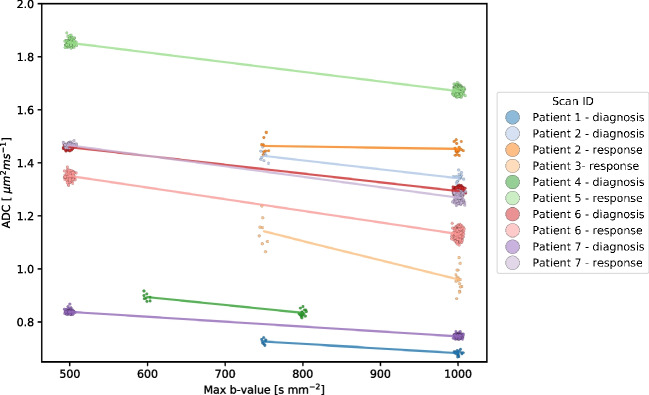
Diffusion-weighted imaging (DWI) using at least six *b*-values was performed in ten scans of seven pediatric rhabdomyosarcoma patients. For each scan, the median apparent diffusion coefficient (ADC) was estimated for the tumor with different combinations of *b*-values. Each combination included the non-weighted diffusion image (*b*=0 s mm^−2^). Examples of *b*-value combinations include: *b*=0, 100, 500, and 1,000 s mm^−2^; *b*=0, 200, and 500 s mm^−2^. The *figure* shows tumor ADC versus the highest *b*-value used to estimate the ADC. For each scan (represented by different *colors*), ADC was calculated with combinations of *b*-values. These are plotted along the *x*-axis based on the highest *b*-value that was used. The ADC trends downwards when higher *b*-values are applied to the acquisition. Averaged over all ten scans, increasing the highest *b*-value by 100 s mm^−2^ results in a decrease in ADC of 2.8%

In a subset of ten examinations with available DW data and more than five *b*-values, we simulated the effect of variation by estimating the ADC with all possible *b*-value combinations. In this cohort, there is a general trend of decreasing ADC with increasing maximum *b*-value (Fig. 6). When averaging over all acquisitions with more than five *b*-values, different choices of the highest *b*-value led to a 2.8% change in the estimated ADC per 100 s mm^−2^. The effect of varying other *b*-values is negligible.

**Fig. 7:**
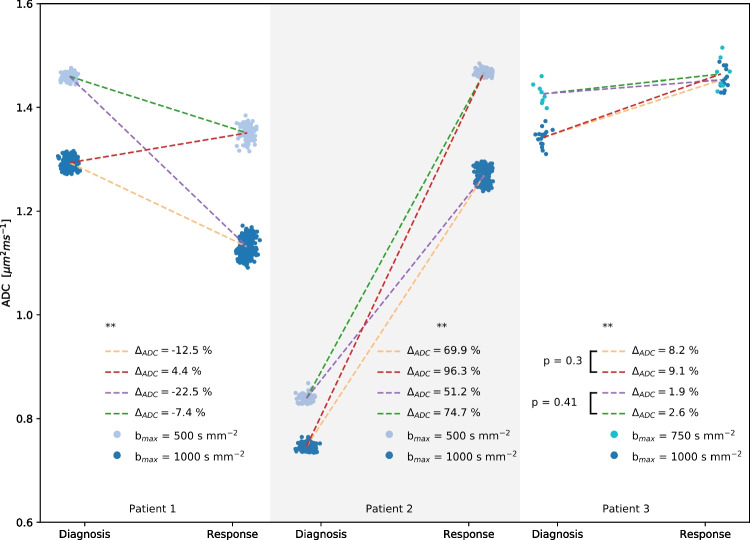
Apparent diffusion coefficient (ADC) estimates of three rhabdomyosarcoma imaged at diagnosis and after chemotherapy with multiple *b*-values. Each *dot* represents a different combination of *b*-values. All combinations include *b*=0 s mm^−2^ and either the highest or second-highest *b*-value that was used in the acquisition. Grouping these combinations by the highest *b*-value adopted and averaging the ADC values of each group results in two values at both time points leading to four options to calculate the longitudinal ADC change in the tumor lesions, shown in the *figure* by the *broken lines*; highest *b*-value at diagnosis and at treatment response (*yellow*), second-highest *b*-value at diagnosis and at treatment response (*green*), second-highest *b*-value at diagnosis and highest at treatment response (*purple*), highest *b*-value at diagnosis and second highest at treatment response (*red*). **All combinations differ significantly with *P*<0.01 based on a *t*-test, except where otherwise indicated

Three patients had scans at both baseline and chemotherapy response assessment with more than five *b*-values; all acquisition parameters were identical at both time points for all of them. Figure 7 shows the impact of varying the highest *b*-value used on the measured longitudinal change in ADC for these children. By using either the highest *b*-value or the second highest at both time points, there are four ways to calculate the longitudinal change. The impact of changing the highest *b*-value on the longitudinal change in ADC varied per patient, but substantial differences were observed in all three patients: from −23% to 4%, from 51% to 93%, and from 2% to 9%. For patients 1 and 2, all combinations significantly differed (*P*<0.01). For patient 3, two combinations did not significantly vary (*P*=0.3 and *P*=0.4), whereas the rest did (*P*<0.01).

## Discussion

In this retrospective multicenter cohort study of rhabdomyosarcoma, we observed large variations in acquisition parameters within and across different hospitals and between scan sessions for the same patient. We could not detect a relation between acquisition parameters and ADC at a cohort level. These results would lead us to conclude that acquisition parameters might not have a significant influence on tumor ADC in multicenter studies. However, other considerations may confound these results.

Prior studies have shown that DWI can be very sensitive to changes in acquisition, which does not align with our findings [13]. Our results are not consistent with the research conducted by Celik and colleagues, who found a relation among TE, TR, and the number of pre-pulses on ADC in a phantom study [14]. Nevertheless, we do confirm a relation of tumor ADC and the highest *b*-value used in acquisition when looking at a smaller subset of the cohort. We observe that a change of 100 s mm^−2^ in the *b*-value results in a decrease of ADC by 2.8%. This aligns with previous studies which have demonstrated that higher *b*-values result in lower ADC values [24–26]. This finding motivates the creation of guidelines to minimize *b*-value variance in future studies. Alternatively, it could be used to harmonize data where the variance was unavoidable. One potential reason for not detecting any correlation between acquisition and ADC values at a cohort level is a large inherent heterogeneity in the tumors, which may mask any potential effects related to acquisition on ADC values. In support of this theory, we observe a larger variance in tumor ADC values than in the ADC values of healthy muscle regions. Comparing these ranges of ADC values with those reported in existing literature, we find similar results for muscle tissue [27] and (soft-tissue) tumors [28, 29]. This points to the observed variance being primarily influenced by tumor and patient characteristics, which may contribute more to the ADC than any effects from varying acquisition parameters.

If we could more accurately determine the effect of parameter variability on tumor ADC, like we have tried with the *b*-values, it would still be difficult to say whether these effects would be clinically relevant, as the effect of treatment response on tumor ADC is still unknown for pediatric rhabdomyosarcoma. When comparing with other pathologies, we find that ADC tumor response results in approximately 20% difference between response and no response groups [30, 31]. This value is low compared to the ADC variance we observed in our cohort.

If we want to develop ADC as a response marker for pediatric rhabdomyosarcoma, we should strive to minimize additional variability from sources such as acquisition parameters as much as possible, for instance by creating uniform imaging guidelines. The heterogeneity of rhabdomyosarcoma tumor locations presents a large challenge in this context, as different locations each impose constraints on imaging parameters. This could be addressed by grouping locations in categories and creating an imaging model per category.

### Methodological considerations

Being a retrospective cohort study, incomplete data was a common issue in this investigation. We encountered missing acquisition parameter values and datasets without diffusion-weighted images, which affected the robustness of our findings.

Similarly, because of the heterogeneity of the cohort, we included patients in multiple risk categories with inherent differences in treatment, such as the number and intensity of chemotherapy cycles, which could potentially affect our results. Unfortunately, we could not correct for the influence of these factors on tumor ADC, since we did not have a sufficient sample size.

Additionally, some datasets included ADC images but lacked the necessary DW data to generate these images. We therefore introduce another potential source of variability stemming from the algorithm used to convert DW to ADC [32]. The impact of this variability on the ADC estimates in the tumors studied here remains unknown.

Our analysis of the impact of *b*-value on tumor ADC relies upon the assumption that two equal *b*-values would provide the same amount of diffusion weighting. Since the *b*-value is a congregate of multiple other parameters, we cannot be sure that this assumption holds true. Unfortunately, as these other parameters are not listed separately in the DICOM standard, we were not able to look into this further.

Lastly, for a subset of patients, we compared tumor ADC values to those measured in muscle tissue located within the same slice as the tumor. However, due to the heterogeneous nature of primary tumor locations, there was no single tissue type consistently present in all images for comparison. We selected muscle tissue due to its similarity to rhabdomyosarcoma, but due to the limited image quality in the more peripheral image regions, we could only perform this comparison in 16 patients. The small sample size prevented a direct assessment of the effects of acquisition parameters on ADC between healthy and tumor tissue.

## Conclusion

Due to the rarity of rhabdomyosarcoma, the heterogeneity in presentation, and the typical lack of international imaging protocols [33], variance in the acquisition protocol has been largely unavoidable. In this study, we show that the effects of acquisition parameters on tumor ADC estimates may complicate the interpretation of findings in an international multicenter cohort. Especially a mismatch between the highest *b*-value at the acquisition at diagnosis and at response assessment can have relevant impact on the observed longitudinal change in ADC. Therefore, it would be useful to perform such a study in a controlled environment where all aspects of treatment and acquisition are kept as similar as possible. This might give a lower-bound for the variance observed in a larger multicenter study and allow for specific variables, such as the influence of *b*-values on ADC, to be evaluated in larger sample sizes.

DWI is part of a sub-study within the ongoing prospective multinational Frontline and Relapsed Rhabdomyosarcoma (FaR-RMS) clinical trial [34]. The aim of this study, which strives to include approximately 1,700 patients, is to assess if DWI potentially can serve as a new early surrogate marker of outcome in rhabdomyosarcoma. In the prospective FaR-RMS study imaging data will centrally be collected and stored using the Keosys Medical Imaging platform (Keosys, Saint-Herblain, France) and post-processing will be done off-line. For this, a homogeneous dataset is required; we therefore call upon all pediatric radiologists who are involved in imaging rhabdomyosarcoma to follow the previously published imaging guidelines by van Ewijk and colleagues when scanning patients with rhabdomyosarcoma [35, 36].

## Supplementary Information

Below is the link to the electronic supplementary material.

Supplementary file1 (XLSX 21 KB)

Supplementary Figure 2. (PNG 136 KB)

Supplementary file2 (TIFF 31.1 KB)

Supplementary Figure 3. (PNG 577 KB)

High resolution image (TIFF 2.76 mb)

Supplementary file4 (PNG 312 KB)

Supplementary file5 (PNG 223 KB)

Supplementary file6 (PNG 233 KB)

Supplementary file7 (PNG 248 KB)

## Data Availability

No datasets were generated or analysed during the current study.

## References

[1] Van Ewijk R, Vaarwerk B, Breunis WB et al (2021) The value of early tumor size response to chemotherapy in pediatric rhabdomyosarcoma. Cancers 13:510 33561094 10.3390/cancers13030510 PMC7866196

[2] Chen L, Liu M, Bao J et al (2013) The correlation between apparent diffusion coefficient and tumor cellularity in patients: a meta-analysis. PLoS ONE 8:e79008 24244402 10.1371/journal.pone.0079008 PMC3823989

[3] Schnapauff D, Zeile M, Niederhagen MB et al (2009) Diffusion-weighted echo-planar magnetic resonance imaging for the assessment of tumor cellularity in patients with soft-tissue sarcomas. Magnet Resonance Imaging 29:1355–1359 10.1002/jmri.21755 19472392

[4] Humphries PD, Sebire NJ, Siegel MJ, Olsen ØE (2007) Tumors in pediatric patients at diffusion-weighted MR imaging: apparent diffusion coefficient and tumor cellularity. Radiology 245:848–854 17951348 10.1148/radiol.2452061535

[5] Pilatus U, Shim H, Artemov D et al (1997) Intracellular volume and apparent diffusion constants of perfused cancer cell cultures, as measured by NMR. Magnetic Resonance in Med 37:825–832 10.1002/mrm.1910370605 9178232

[6] Jiang R, Ma Z, Dong H et al (2016) Diffusion tensor imaging of breast lesions: evaluation of apparent diffusion coefficient and fractional anisotropy and tissue cellularity. BJR 89:20160076 27302492 10.1259/bjr.20160076 PMC5124883

[7] Moustafa AFI, El Said SSMAS, Moustafa MA et al (2019) Diffusion-weighted MR imaging diagnostic merits in the post-therapeutic assessment of musculoskeletal soft tissue sarcoma. Egypt J Radiol Nucl Med 50:52

[8] Sharma U, Danishad KKA, Seenu V, Jagannathan NR (2009) Longitudinal study of the assessment by MRI and diffusion-weighted imaging of tumor response in patients with locally advanced breast cancer undergoing neoadjuvant chemotherapy. NMR Biomed 22:104–113 18384182 10.1002/nbm.1245

[9] Yuan Y, Zeng D, Zhang Y et al (2020) Intravoxel incoherent motion diffusion-weighted imaging assessment of microvascular characteristics in the murine embryonal rhabdomyosarcoma model. Acta Radiol 61:260–266 31226880 10.1177/0284185119855731

[10] Thoeny HC, De Keyzer F, Vandecaveye V et al (2005) Effect of vascular targeting agent in rat tumor model: dynamic contrast-enhanced versus diffusion-weighted MR imaging. Radiology 237:492–499. 10.1148/radiol.2372041638 16192323 10.1148/radiol.2372041638

[11] Afaq A (2010) Diffusion-weighted magnetic resonance imaging for tumour response assessment: why, when and how? Cancer Imaging 10:S179–S188 20880779 10.1102/1470-7330.2010.9032 PMC2967137

[12] Messina C, Bignone R, Bruno A et al (2020) Diffusion-weighted imaging in oncology: an update. Cancers 12:1493 32521645 10.3390/cancers12061493 PMC7352852

[13] Padhani AR, Liu G, Mu-Koh D et al (2009) Diffusion-weighted magnetic resonance imaging as a cancer biomarker: consensus and recommendations. Neoplasia 11:102–125 19186405 10.1593/neo.81328 PMC2631136

[14] Celik A (2015) Effect of imaging parameters on the accuracy of apparent diffusion coefficient and optimization strategies. Diagn Interv Radiol 22:101–107 10.5152/dir.2015.14440 PMC4712890 26573977

[15] Schmeel FC (2019) Variability in quantitative diffusion-weighted MR imaging (DWI) across different scanners and imaging sites: is there a potential consensus that can help reducing the limits of expected bias? Eur Radiol 29:2243–2245 30488105 10.1007/s00330-018-5866-4

[16] Kaatsch P (2010) Epidemiology of childhood cancer. Cancer Treat Rev 36:277–285 20231056 10.1016/j.ctrv.2010.02.003

[17] Ward E, DeSantis C, Robbins A et al (2014) Childhood and adolescent cancer statistics, 2014. CA A Cancer J Clinic 64:83–103 10.3322/caac.21219 24488779

[18] Van Ewijk R, Chatziantoniou C, Adams M et al (2023) Quantitative diffusion-weighted MRI response assessment in rhabdomyosarcoma: an international retrospective study on behalf of the European Paediatric Soft Tissue Sarcoma Study Group Imaging Committee. Pediatr Radiol 53:2539–2551 37682330 10.1007/s00247-023-05745-z PMC10635937

[19] Bisogno G, De Salvo GL, Bergeron C et al (2019) Vinorelbine and continuous low-dose cyclophosphamide as maintenance chemotherapy in patients with high-risk rhabdomyosarcoma (RMS 2005): a multicentre, open-label, randomised, phase 3 trial. Lancet Oncol 20:1566–1575 31562043 10.1016/S1470-2045(19)30617-5

[20] Bisogno G, Minard-Colin V, Zanetti I et al (2023) Nonmetastatic rhabdomyosarcoma in children and adolescents: overall results of the European Pediatric Soft Tissue Sarcoma Study Group RMS2005 Study. JCO 41:2342–2349 10.1200/JCO.22.02093 36848614

[21] Schoot RA, Chisholm JC, Casanova M et al (2022) Metastatic rhabdomyosarcoma: results of the European Paediatric Soft Tissue Sarcoma Study Group MTS 2008 Study and pooled analysis with the concurrent BERNIE Study. JCO 40:3730–3740 10.1200/JCO.21.02981 PMC9649279 35709412

[22] Kelly SM, Effeney R, Gaze MN et al (2022) QUARTET: a SIOP Europe project for quality and excellence in radiotherapy and imaging for children and adolescents with cancer. Eur J Cancer 172:209–220 35780527 10.1016/j.ejca.2022.05.037

[23] Chatziantoniou C, Schoot RA, Van Ewijk R et al (2023) Methodological considerations on segmenting rhabdomyosarcoma with diffusion-weighted imaging—what can we do better? Insights Imaging 14:19 36720720 10.1186/s13244-022-01351-z PMC9889596

[24] Tamura T, Murakami S, Naito K et al (2014) Investigation of the optimal b-value to detect breast tumors with diffusion weighted imaging by 1.5-T MRI. Cancer imaging 14:11 25608450 10.1186/1470-7330-14-11 PMC4331817

[25] Peters NHGM, Vincken KL, Van Den Bosch MAAJ et al (2010) Quantitative diffusion weighted imaging for differentiation of benign and malignant breast lesions: the influence of the choice of *b* -values. Magnet Resonance Imaging 31:1100–1105 10.1002/jmri.22152 20432344

[26] Dale BM, Braithwaite AC, Boll DT, Merkle EM (2010) Field strength and diffusion encoding technique affect the apparent diffusion coefficient measurements in diffusion-weighted imaging of the abdomen. Invest Radiol 45:104–108 20027117 10.1097/RLI.0b013e3181c8ceac

[27] Schlaffke L, Rehmann R, Rohm M et al (2019) Multi-center evaluation of stability and reproducibility of quantitative MRI measures in healthy calf muscles. NMR Biomed 32:e4119 31313867 10.1002/nbm.4119

[28] Surov A, Nagata S, Razek AAA et al (2015) Comparison of ADC values in different malignancies of the skeletal musculature: a multicentric analysis. Skeletal Radiol 44:995–1000 25916616 10.1007/s00256-015-2141-5

[29] Nakajo M, Fukukura Y, Hakamada H et al (2018) Whole-tumor apparent diffusion coefficient (ADC) histogram analysis to differentiate benign peripheral neurogenic tumors from soft tissue sarcomas. Magnet Reson Imaging 48:680–686 10.1002/jmri.25987 29469942

[30] Blažić I, Maksimović R, Gajić M, Šaranović Đ (2015) Apparent diffusion coefficient measurement covering complete tumor area better predicts rectal cancer response to neoadjuvant chemoradiotherapy. Croat Med J 56:460–469 26526883 10.3325/cmj.2015.56.460 PMC4655931

[31] Vollenbrock SE, Voncken FEM, Bartels LW et al (2020) Diffusion-weighted MRI with ADC mapping for response prediction and assessment of oesophageal cancer: a systematic review. Radiother Oncol 142:17–26 31431376 10.1016/j.radonc.2019.07.006

[32] Taouli B, Beer AJ, Chenevert T et al (2016) Diffusion-weighted imaging outside the brain: consensus statement from an ISMRM-sponsored workshop. Magnet Reson Imaging 44:521–540 10.1002/jmri.25196 PMC4983499 26892827

[33] Schoot RA, Shulkin BL, Van Rijn RR, Von Kalle T (2023) Pediatric rhabdomyosarcoma protocols should include more detailed imaging guidelines to ensure homogeneous response assessment. JCO 41:2337–2341 10.1200/JCO.22.02613 36758191

[34] Chisholm J, Mandeville H, Adams M et al (2024) Frontline and Relapsed Rhabdomyosarcoma (FaR-RMS) Clinical Trial: a report from the European Paediatric Soft Tissue Sarcoma Study Group (EpSSG). Cancers 16:998 38473359 10.3390/cancers16050998 PMC10931395

[35] Van Ewijk R, Schoot RA, Sparber-Sauer M et al (2021) European guideline for imaging in paediatric and adolescent rhabdomyosarcoma — joint statement by the European Paediatric Soft Tissue Sarcoma Study Group, the Cooperative Weichteilsarkom Studiengruppe and the Oncology Task Force of the European Society of Paediatric Radiology. Pediatr Radiol 51:1940–1951 34137936 10.1007/s00247-021-05081-0 PMC8426307

[36] Schoot RA, Van Ewijk R, Von Witzleben A-A et al (2023) INternational Soft Tissue saRcoma ConsorTium (INSTRuCT) consensus statement: imaging recommendations for the management of rhabdomyosarcoma. Eur J Radiol 166:111012 37541182 10.1016/j.ejrad.2023.111012

