# Supplementary figures and images for: Acquisition matters - how do scan parameters affect apparent diffusion coefficient estimates in pediatric rhabdomyosarcoma
Source: Pediatr Radiol. 2025 Jun 10;55(8):1598–610. doi: 10.1007/s00247-025-06263-w (PMC12321683; doi:10.1007/s00247-025-06263-w)

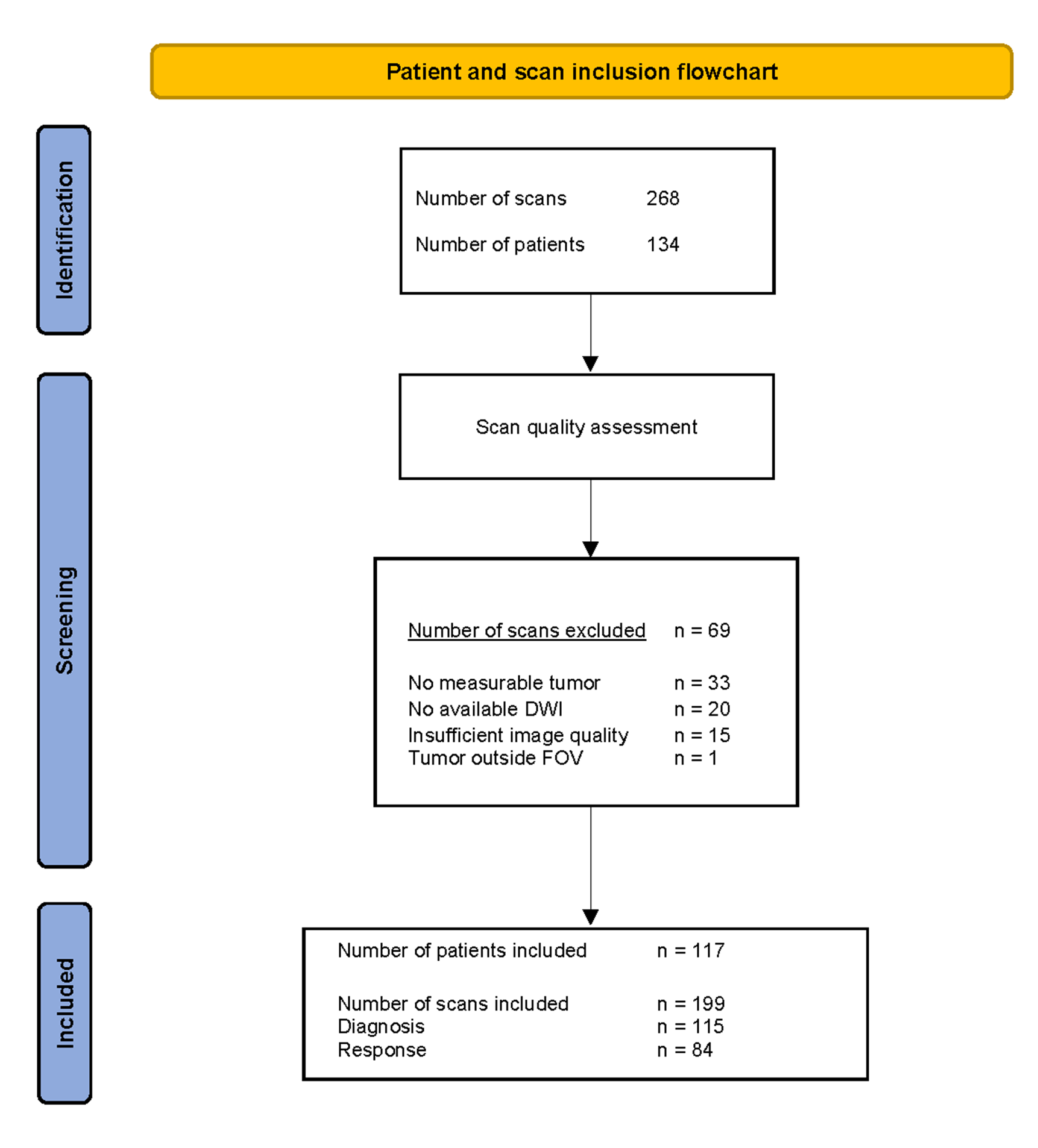

Supplement: Supplementary file 2 — (PNG 136 KB) [file 247_2025_6263_Fig8_ESM.png]

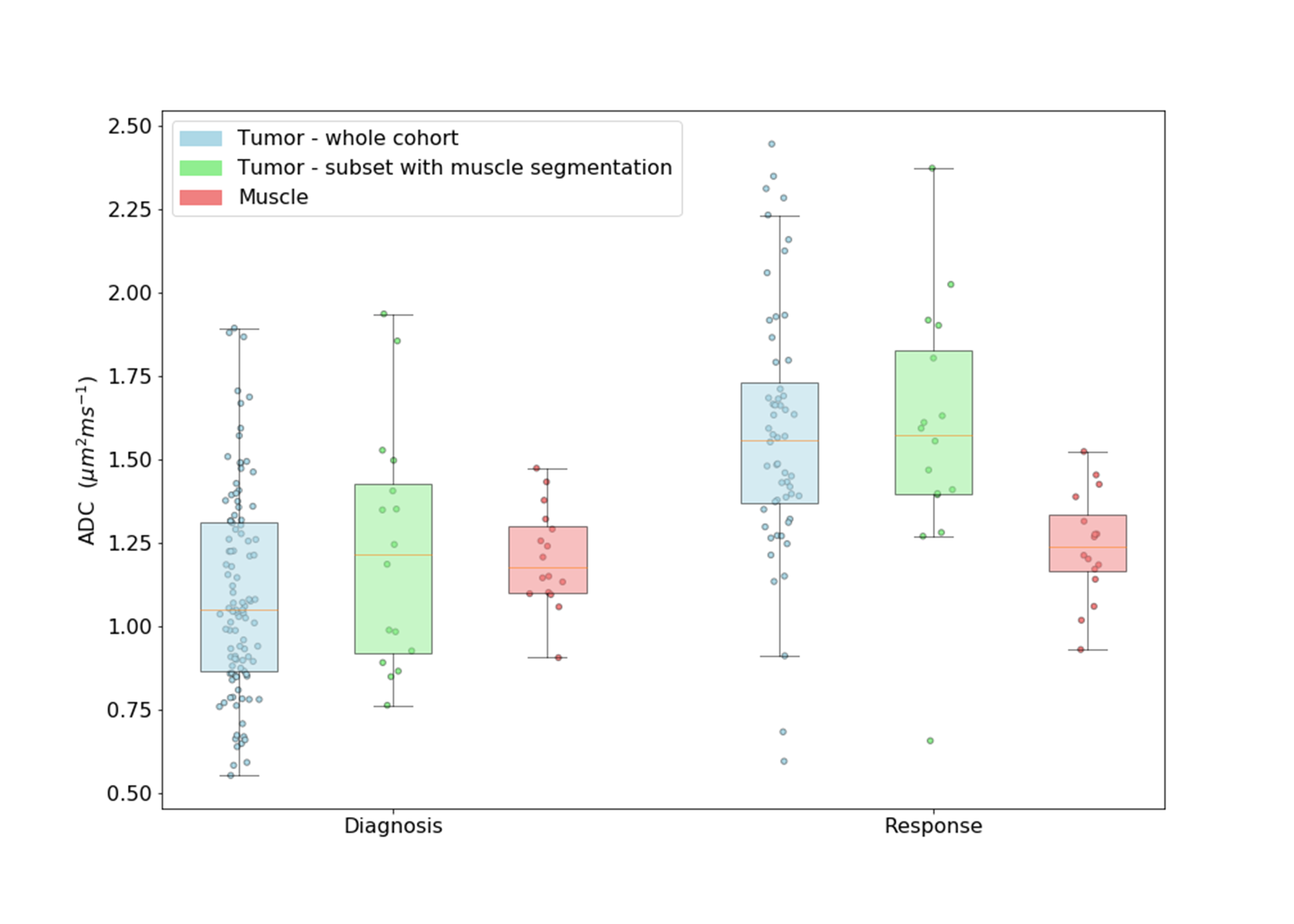

Supplement: Supplementary file 4 — (PNG 577 KB) [file 247_2025_6263_Fig9_ESM.png]

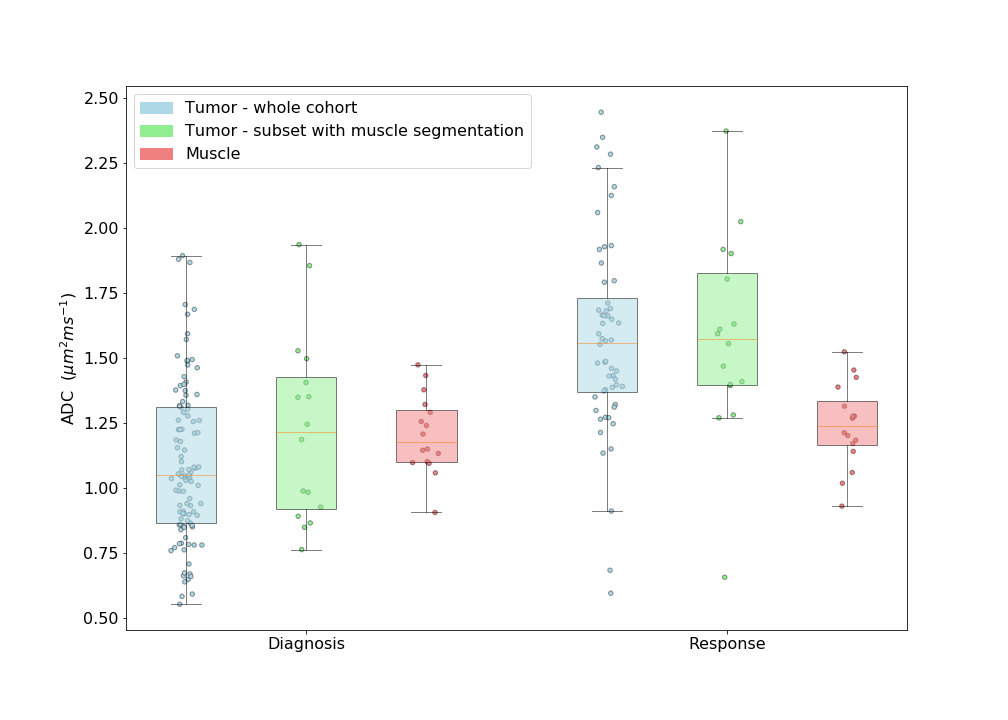

Supplement: Supplementary file 5 — High resolution image (TIFF 2.76 mb) [file 247_2025_6263_MOESM3_ESM.tiff]

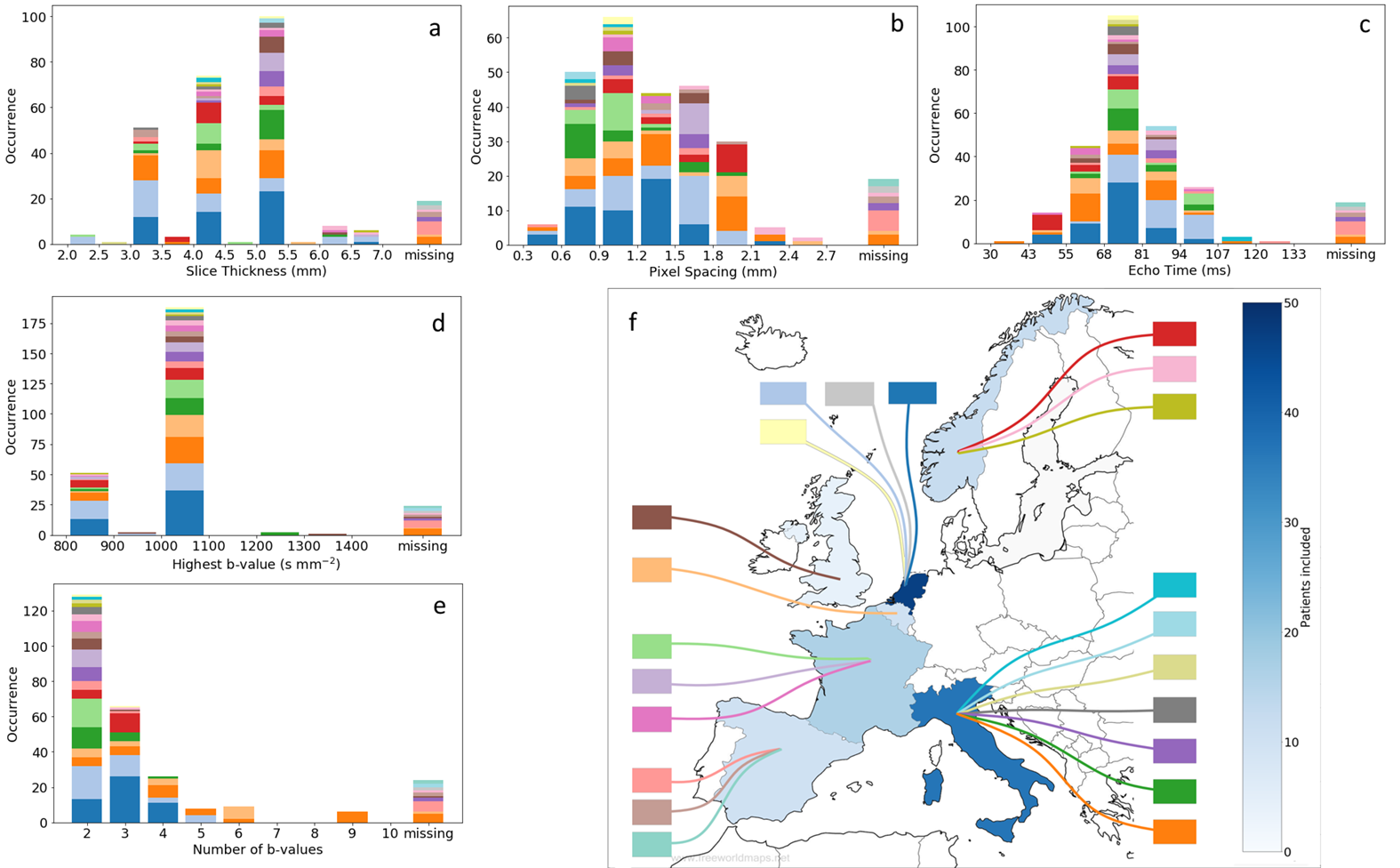

Supplement: Supplementary file 6 — Supplementary file4 (PNG 312 KB) [file 247_2025_6263_MOESM4_ESM.png]

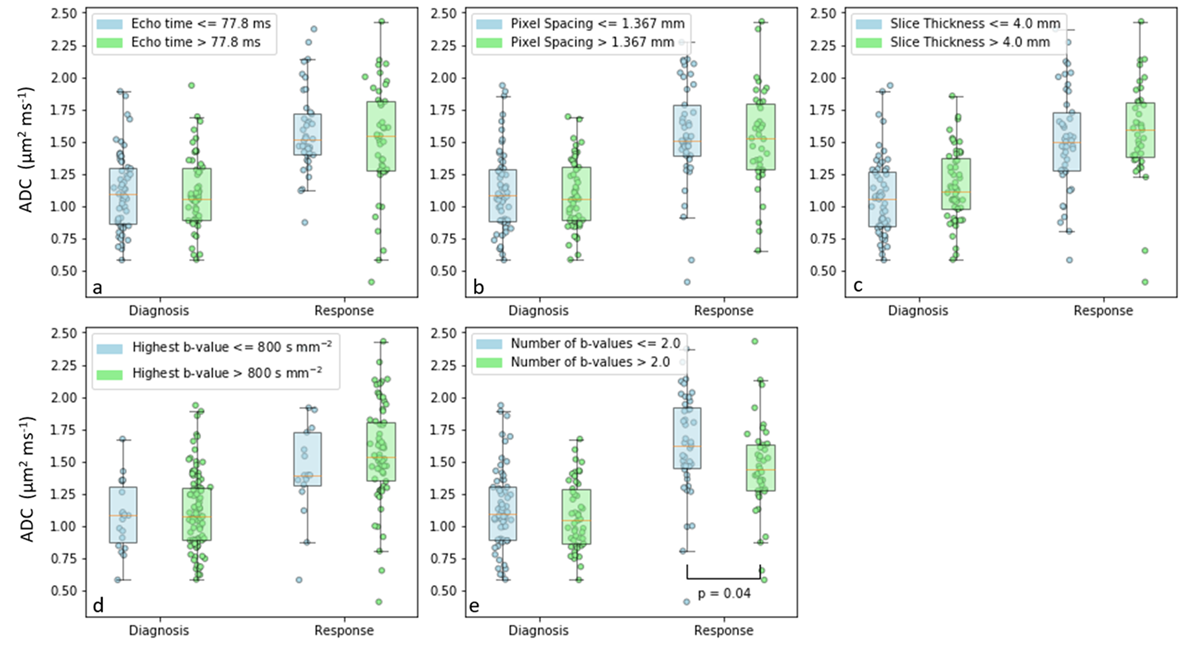

Supplement: Supplementary file 7 — Supplementary file5 (PNG 223 KB) [file 247_2025_6263_MOESM5_ESM.png]

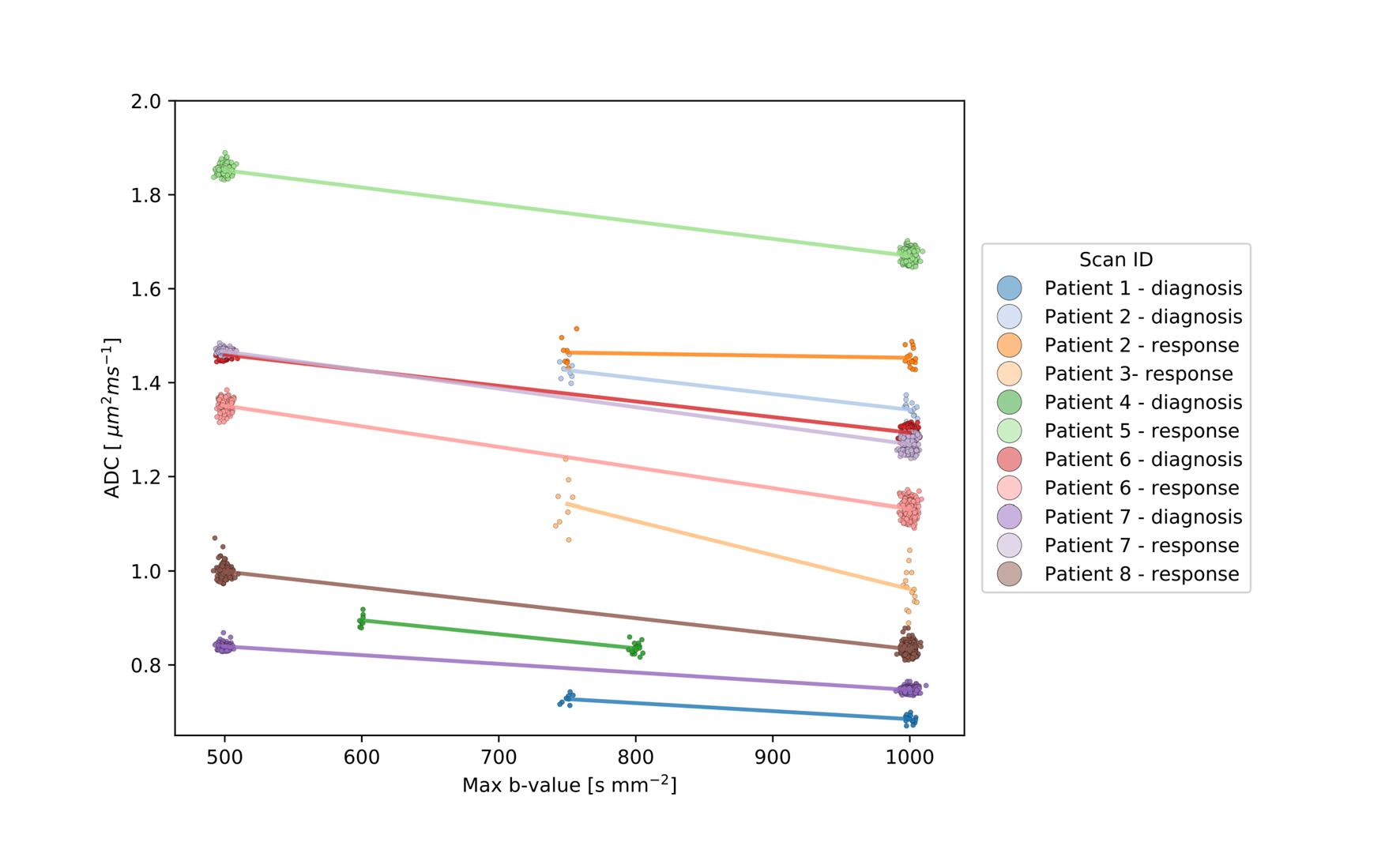

Supplement: Supplementary file 8 — Supplementary file6 (PNG 233 KB) [file 247_2025_6263_MOESM6_ESM.png]

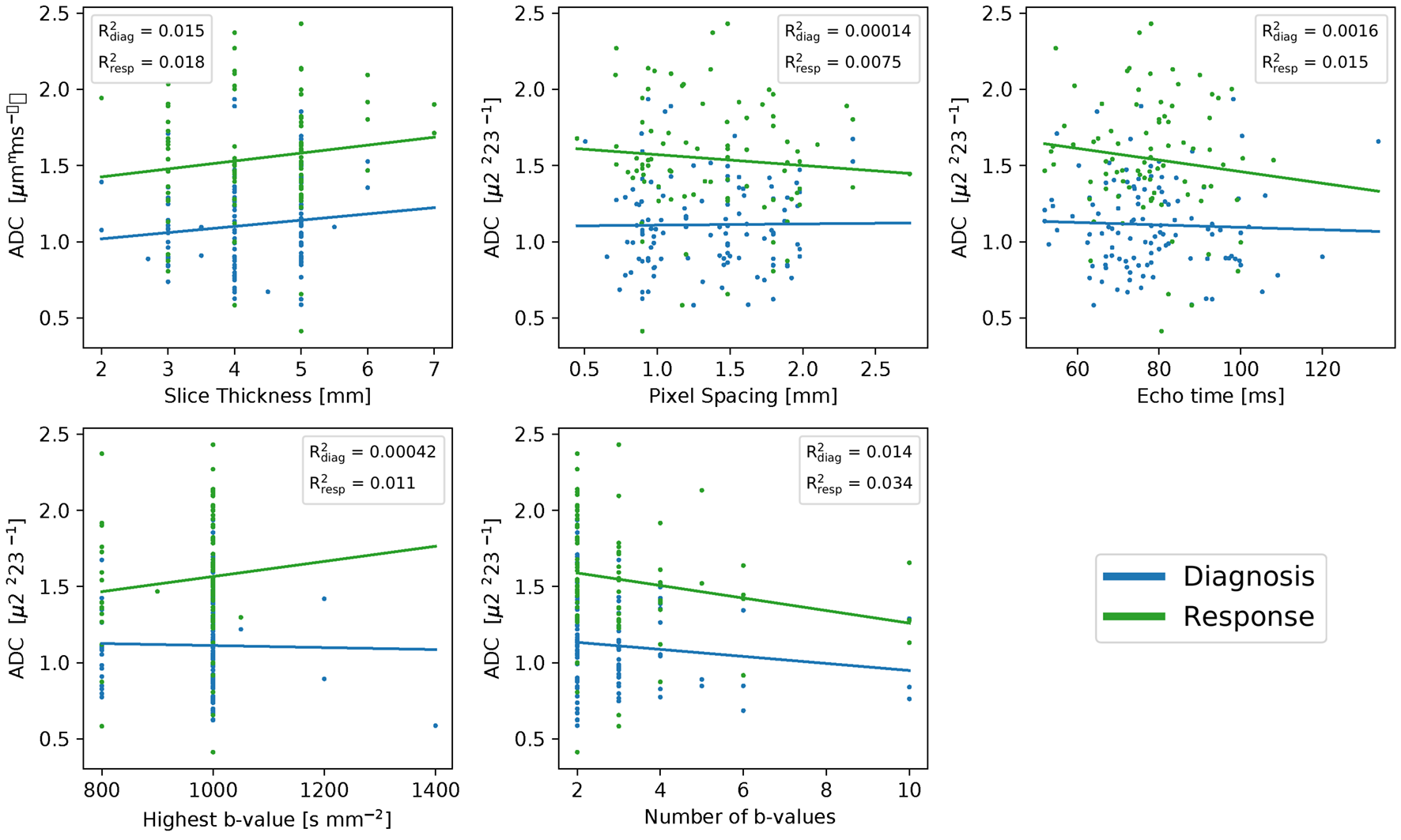

Supplement: Supplementary file 9 — Supplementary file7 (PNG 248 KB) [file 247_2025_6263_MOESM7_ESM.png]
